# Influence of the Template Removal Method on the Mechanical Stability of SBA‐15

**DOI:** 10.1002/open.202100225

**Published:** 2021-11-05

**Authors:** Ann‐Katrin Beurer, Johanna R. Bruckner, Yvonne Traa

**Affiliations:** ^1^ Institute of Technical Chemistry University of Stuttgart 70550 Stuttgart Germany; ^2^ Institute of Physical Chemistry University of Stuttgart 70550 Stuttgart Germany

**Keywords:** calcination, mechanical stability, ordered mesoporous silica, SBA-15, Soxhlet extraction

## Abstract

Removing the template from the pores after the polycondensation of the silica precursor is a necessary step in the synthesis of mesoporous silica materials. In our previous work, we developed a method for the efficient and spatially controlled functionalization of SBA‐15. First, the silanol groups on the particle surface and in the pore entrances were passivated. After extraction of the template, a pretreatment step in N_2_ converted the silanol groups to the single and geminal state. Afterwards, an azide functionality was introduced exclusively into the mesopores. This ensured that the catalyst could afterwards be immobilized unambiguously in the mesopores. The mechanical stability of a material functionalized in such a spatially controlled manner is studied and compared to other template removal methods. Even though several studies investigated the influence of the calcination temperature, the presence or the absence of oxygen during the template removal, the specific conditions used during the herein reported selective functionalization procedure have not been covered yet.

## Graphical abstract

**Removing the template** from mesoporous SBA‐15 by calcination in air was evaluated against Soxhlet extraction with ethanol, followed by pretreatment at 550 °C in N_2_, with regard to the mechanical stability of the materials. Pressures of 39 and 156 MPa were applied. The results are relevant for the stepwise selective functionalization, as the removal of the template without a calcination step allows for a more time‐ and material‐efficient functionalization procedure.

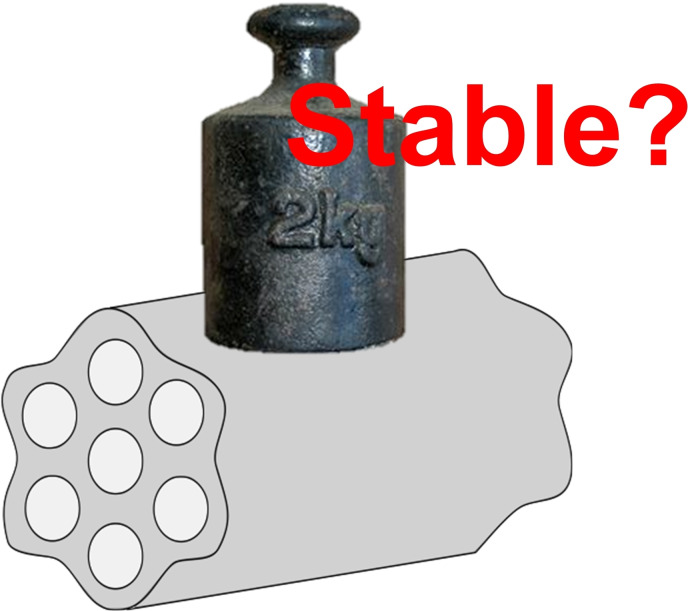

Mesoporous silica materials modified with different functionalities can be used in heterogeneous catalysis.[^1^, ^2^] Spatially controlled functionalization of porous materials is necessary for the study of confinement effects in catalytic applications. For this reason, it is important that the developed catalysts can be used in larger reactors and synthesized on a larger scale. Therefore, the catalysts, which are often prepared in powder form, must be formed, for example, by tableting or extrusion. On that account, the mechanical stability of the catalysts is of interest. In the various approaches to the selective functionalization of mesoporous materials, different template removal methods are used.[^3^, ^4^, ^5^, ^6^] However, the question if the template removal method affects the mechanical stability of the mesoporous support material has not been addressed so far. One method for the selective functionalization starts with calcined mesoporous silica material, which is commercially available. In this case, it is necessary to refill the pores with template before the functionalization of the particle surface. Soxhlet extraction with ethanol reopens the pores after the functionalization. In the last step, the pore walls can be modified.^[7]^ Another method, which we disclosed in a previous report,^[8]^ is more time‐ and material‐efficient, as the particle surface of mesoporous silica is functionalized prior to the removal of the template. In a second step, Soxhlet extraction with ethanol and thermal treatment in N_2_ open the pores and activate the silanol groups. The pretreatment is carried out in N_2_ to protect the organic groups on the particle surface. If the thermal treatment is carried out in air, the organic groups would burn and thus no longer ensure that the catalytically active component would only be present in the pores of the support material after the functionalization step.[^9^, ^10^] Additionally, this manufacturing process also opens the possibility to recycle the structure‐directing template. Subsequently, the pore walls can be functionalized. The conditions during the functionalization steps are comparatively gentle compared to the ones during the template removal and should not affect the mechanical stability of the mesoporous material.

Up to now, studies reported in the literature have dealt with the influence of the calcination temperature,[^11^, ^12^] the presence or absence of oxygen during the template removal,[^13^, ^14^] and with the comparison of different porous silica materials with regard to their mechanical stability.[^15^, ^16^, ^17^] Furthermore, the mechanical stability of calcined SBA‐15 as a function of pressures between 16 and 260 MPa has been considered.[^17^, ^18^] The XRD diffractograms showed a loss of intensity of the characteristic reflections (100), (110) and (200) for the calcined and pressed SBA‐15. The decrease of the intensities was attributed to a loss of the long‐range order. The lattice parameters and the main pore size of the different samples did not change compared to the unpressed material. However, the pore size distribution widened with increasing pressure. The authors explained this by a deformation of the pores during pressing. Due to the change of the pores, the surface area and the mesopore volume decreased, too.[^17^, ^18^] As preliminary investigations for this communication, we determined the mechanical stability of calcined SBA‐15 (**SBA‐15‐calc**; for an explanation of the nomenclature, see Supporting Information chapter 1) against pressures similar to what has been reported in the literature (see Supporting Information, chapter 2). Our results concerning small angle X‐ray scattering (SAXS) and N_2_ physisorption measurements are in agreement with the literature.[^17^, ^18^]

Based on the insights from these investigations, we considered the influence of the template removal method. For this purpose, we investigated and compared the intermediates of the selective functionalization process with each other. The selective functionalization of the surfaces of SBA‐15 was omitted (see Supporting Information, chapter 3), because it occurs in every method and is known to further increase the mechanical stability.^[15]^ The materials under study encompass **SBA‐15‐calc**, which was calcined in air, and calcined SBA‐15 whose pores were first refilled with the template (Pluronic® P‐123) and then reopened by Soxhlet extraction with ethanol (**SBA‐15‐calc‐re‐E**). In addition, SBA‐15 whose pores were opened by Soxhlet extraction with ethanol (**SBA‐15‐as‐E**) and SBA‐15 heated to 400 °C or 550 °C in N_2_ after Soxhlet extraction (**SBA‐15‐as‐E**‐**p400** and **SBA‐15‐as‐E‐p550**) were considered. All SBA‐15 samples were then pressed into a tablet for 10 min with a pressure of 156 MPa and carefully crushed for further investigations (appended with “156 MPa”).

The SAXS experiments show that the hexagonal lattice parameter (Figure 1, Table 1) does not change significantly upon pressing compared to the unpressed materials (see Supporting Information, Figures S5–S8). The SAXS curves of **SBA‐15‐calc‐156MPa** and **SBA‐15‐calc‐re‐E‐156MPa** are almost identical. This can be explained by the fact that the calcination conditions are much harsher than those during Soxhlet extraction with ethanol. Therefore, calcination is expected to have a greater effect on the structure. To prove this assumption, the SAXS curves of **SBA‐15‐as**, **SBA‐15‐calc** and **SBA‐15‐as‐E** must be considered (Supporting Information, Figures S4, S5, S8). The reflexes in the SAXS curves of **SBA‐15‐calc** show a shift to smaller values of the scattering vector, resulting in a smaller lattice parameter compared to the lattice parameter of **SBA‐15‐as** (Tables 1, S4). This indicates that the distance from pore center to pore center is smaller for **SBA‐15‐calc**. The described effect is not observable for **SBA‐15‐as‐E**. Therefore, it can be assumed that calcination has a greater effect on the structure of **SBA‐15** than Soxhlet extraction with ethanol. In the synthesis of **SBA‐15‐calc‐re‐E**, a combination of both methods is used to remove the template. However, since calcination is performed first and then ethanol extraction is performed after refilling the pores with P‐123, it can be assumed that the influence of calcination is much larger and the structure as well as the mechanical stability properties of **SBA‐15‐calc‐re‐E** are more similar to those of **SBA‐15‐calc**. Accordingly, it is also expected that the intensities of the SAXS curves of **SBA‐15‐calc‐re‐E‐156MPa** and **SBA‐15‐calc‐156MPa** are identical or very similar. **SBA‐15‐as‐E** and **SBA‐15‐as‐E‐156MPa** exhibit a lattice parameter value of *a*=12.2 nm, while the lattice parameters of the samples treated at 550 °C in N_2_ are significantly smaller, suggesting a degree of silica condensation similar to the one after calcination.

**Figure 1:**
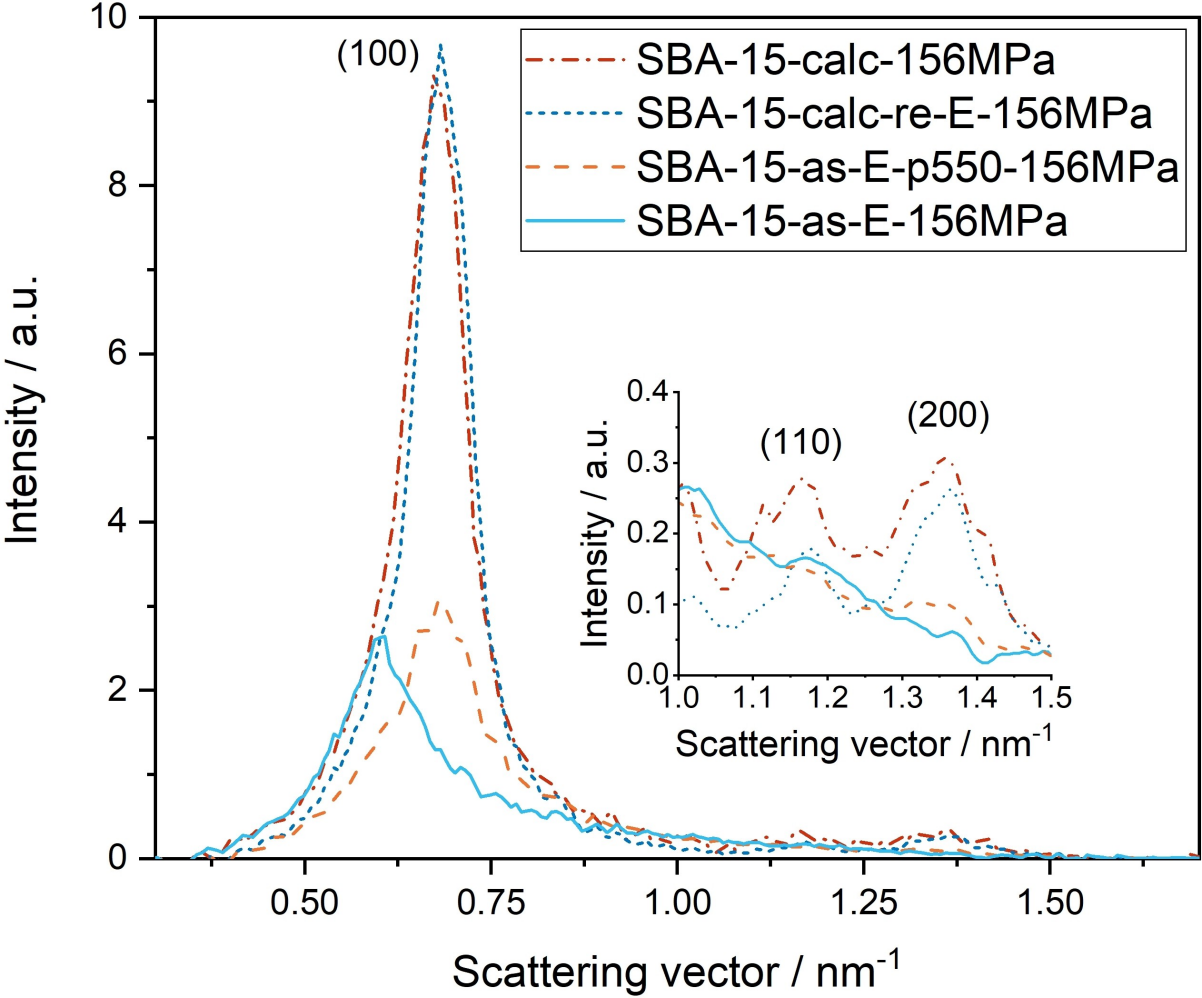
SAXS curves of the samples **SBA‐15‐calc**, **SBA‐15‐calc‐re‐E**, **SBA‐15‐as‐E** and **SBA‐15‐as‐E‐p550**, which were all pressed with 156 MPa for 10 min.

**Table 1:** Total surface determined by the BET method (*S*_BET_), micropore surface (*S*_micro_) as well as the total volume (*V*_tot_), the mesopore volume (*V*_meso_) and the micropore volume (*V*_micro_) of differently treated SBA‐15 samples and their analogues pressed with 39 MPa or 156 MPa. Furthermore, the pore diameters determined by the DFT method (*d*_pore,DFT_), the lattice parameter (*a*) from the SAXS measurements and the percentage of removed template (Δ) calculated (for details see Supporting Information, chapter 4) from results of the elemental analysis are listed.

Sample name	*S*_BET_ [m^2^ g^−1^]	*S*_micro_ [m^2^ g^−1^]	*V*_tot_ [cm^3^ g^−1^]	*V*_meso_ [cm^3^ g^−1^]	*V*_micro_ [cm^3^ g^−1^]	*d*_pore,DFT_ [nm]	*a* [nm]	Δ [%]
**SBA‐15‐calc**	897	222	1.128	1.033	0.095	7.0	10.9	97
**SBA‐15‐calc‐39MPa**	772	193	0.976	0.897	0.079	7.0	10.9	n.d.^[a]^
**SBA‐15‐calc‐156MPa**	670	125	0.948	0.899	0.049	6.8	10.9	n.d.^[a]^
**SBA‐15‐calc‐re‐E**	510	0	1.345	1.345	0	6.8	10.7	62
**SBA‐15‐calc‐re‐E‐156MPa**	409	7	0.699	0.699	0	6.6	10.8	n.d.^[a]^
**SBA‐15‐as‐E**	727	134	1.094	1.042	0.052	7.6	12.2	15
**SBA‐15‐as‐E‐156MPa**	460	170	0.371	0.299	0.072	2.6	12.2	n.d.^[a]^
**SBA‐15‐as‐E‐p550**	869	236	1.148	1.053	0.095	7.0	11.1	99
**SBA‐15‐as‐E‐p550‐39MPa**	819	222	0.995	0.902	0.093	6.8	11.0	n.d.^[a]^
**SBA‐15‐as‐E‐p550‐156MPa**	720	209	0.697	0.610	0.087	6.8	10.8	n.d.^[a]^

[a] The pressed samples were not investigated by elemental analysis. It can be assumed that no change in the carbon content has occurred as a result of pressing. Accordingly, the amount of removed Pluronic® P‐123 corresponds to that of the unpressed material.

Looking at the diffractograms in Figure 1, it however becomes obvious that the similar lattice parameter is not a good indicator for the stability of the materials. While the pressure has only a minor effect on the two calcined samples, both **SBA‐15‐as‐E‐156MPa** and **SBA‐15‐as‐E‐p550‐156MPa** show a partial corruption of the nanostructure as revealed by the intensity decrease and the broadening of the scattering peaks. A direct connection between the intensity decrease and the structural integrity cannot be drawn easily, as discussed in detail in the Supporting Information. The widths of the scattering maxima, though, correlate with the degree of order in the two‐dimensional hexagonal lattice as described by the paracrystal model.^[19]^ Comparing the width increase of the (100) peaks of these two samples before and after applying pressure (Figures S7, S8) suggests that the corruption of the structure is stronger for **SBA‐15‐as‐E‐156MPa** (+190 %) than for **SBA‐15‐as‐E‐p550‐156MPa** (+72 %).

Furthermore, N_2_ physisorption measurements were performed (Figure 2). **SBA‐15‐calc** shows a type IV isotherm with the H1 hysteresis typical for mesoporous materials (Figure 2(a)).^[21]^ After pressing at 156 MPa, a type IV isotherm is still present, but the H1 hysteresis is weakened. This change suggests that the uniformity of the pores is lost due to the pressure.^[11]^ This assumption of the corrupted structure is confirmed by the pore size distributions. While **SBA‐15‐calc** shows a sharp pore size distribution with a main pore diameter of 7.0 nm and only few pores in the microporous region, **SBA‐15‐calc‐156MPa** shows a broadening of the pore size distribution and a loss of pores with the original main pore diameter. This is also confirmed by the surface areas and pore volumes listed in Table 1. For **SBA‐15‐calc‐re‐E**, similar results were found (Figure 2(b), Table 1). However, the BET surface areas are significantly lower compared to the solely calcined samples, and no micropores seem to be present. The minimal increase in micropore surface area for samples whose last treatment step was the removal of the template by extraction is due to the error of the measurement method. Furthermore, elemental analysis showed that Pluronic® P‐123 cannot be removed by Soxhlet extraction with ethanol alone (Tables 1, S3). Accordingly, parts of the pore system, especially the micropores, remain closed and are thus inaccessible to N_2_ during the physisorption measurement, causing an underestimation of the micropore surface area. Most likely, upon pressing, parts of these micropores are freed from the template or micropores become accessible by breaking the pore walls, which leads to an apparent increase of the micropore surface area. Even though there is residual template in the pores of **SBA‐15‐calc‐re‐E‐156MPa**, the broadening of the pore size distribution illustrates that it has no supporting and thus no positive influence on the mechanical stability of the material against pressure.

**Figure 2:**
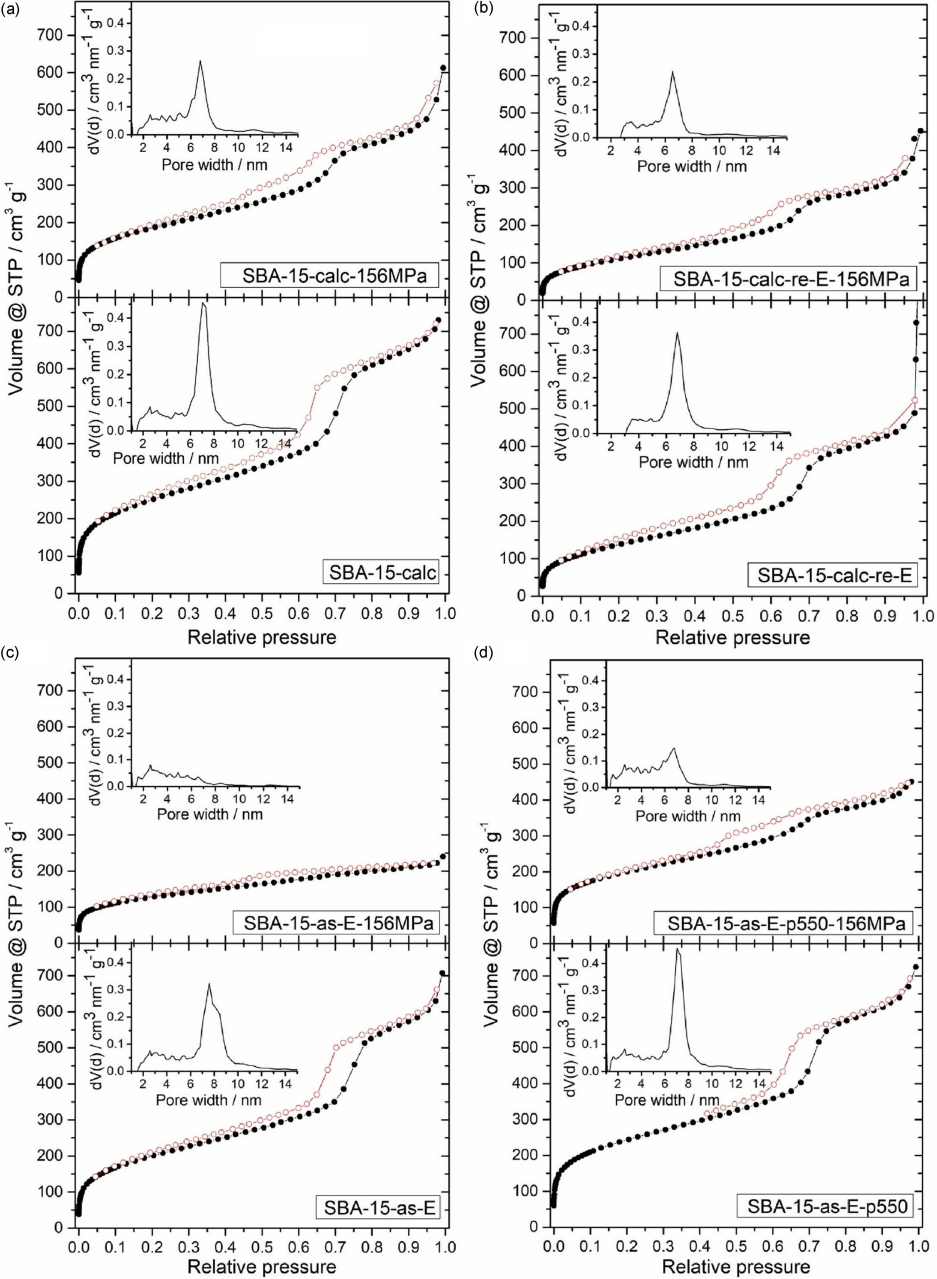
N_2_ adsorption (•) and desorption (○) isotherms and pore size distributions of (a) **SBA‐15‐calc**, (b) **SBA‐15‐calc‐re‐E**, (c) **SBA‐15‐as‐E** and (d) **SBA‐15‐as‐E‐p550** and their analogues pressed with 156 MPa for 10 min.

Knowing that calcination might repair imperfections in the lattice of **SBA‐15‐as**,^[11]^ we anticipated that the silica lattice of **SBA‐15‐calc** should be more stable than the one of **SBA‐15‐as‐E. SBA‐15‐as‐E** also shows a typical type IV isotherm with H1 hysteresis (Figure 2(c)). Pressing at 156 MPa changes the hysteresis to a H4 hysteresis, indicating a complex pore structure.^[21]^ The pore size distributions, the surface areas and pore volumes calculated from the N_2_ physisorption isotherms confirm the destructive effect of the pressure (Table 1). For **SBA‐15‐as‐E‐p550‐156MPa**, we found that the isotherm, the pore size distribution, the calculated surface areas and pore volumes are similar to those of **SBA‐15‐calc** and **SBA‐15‐calc‐re‐E** (Figure 2, Table 1). The hysteresis of **SBA‐15‐as‐E‐p550‐156MPa** is very similar to that of **SBA‐15‐calc‐156MPa** and is much more pronounced than for **SBA‐15‐as‐E‐156MPa**. This behavior can be explained by the elevated temperature during the pretreatment step in N_2_ which leads to further condensation reactions in the SiO_2_ lattice.^[11]^ This is expected to be accompanied by an increase in stability of the pore walls of **SBA‐15‐as‐E‐p550** in comparison to **SBA‐15‐as‐E**. Furthermore, it leads to reduction of the lattice parameter to a similar value as found for **SBA‐15‐calc** (Table 1). While the pore size distributions of **SBA‐15‐as‐E‐p550** and **SBA‐15‐calc** are almost identical, pressing of **SBA‐15‐as‐E‐p550** at 156 MPa results in a greater loss of pores with the main pore diameter than in the case of **SBA‐15‐calc**, indicating that calcination in air leads to slightly more stable materials.

In a continuative experiment, we investigated the influence of the temperature during the thermal treatment and found that the increase in stability correlates with the absolute value of the temperature, that is, the materials treated in N_2_ at 550 °C are more stable than the ones treated in N_2_ at 400 °C (see Supporting Information, chapter 6). Additionally, the thermal treatment in N_2_ has the benefit of removing remnants of the template. Due to this, 99 % of the Pluronic® P‐123 are removed from the pores of **SBA‐15‐as‐E‐p550**, whereas only a 62 % removal is found for **SBA‐15‐calc‐re‐E** (Tables 1, S3). The residual template in **SBA‐15‐calc‐re‐E** blocks parts of the pores, leaving less accessible surface area, and might even interfere with catalyzed reactions. In this aspect, **SBA‐15‐as‐E‐p550** is clearly superior.

In laboratory‐scale reactors and ultra‐fast HPLC setups, much lower pressures of up to 40 MPa are used.[^22^, ^23^, ^24^, ^25^, ^26^, ^27^] Thus, we decided to additionally investigate the mechanical stability of **SBA‐15‐calc** and **SBA‐15‐as‐E‐p550** under similar conditions, for which we pressed the samples for 10 min with 39 MPa. The SAXS measurements of **SBA‐15‐calc‐39MPa** and **SBA‐15‐as‐E‐p550‐39MPa** still exhibit the three characteristic (100), (110) and (200) peaks (Figure 3). In both cases, the full width at half maximum of the (100) peaks increases by only 6 % compared to the respective unpressed samples, showing that the damage caused by the applied pressure is the same for both materials and only minor. The intensity changes are due to two conflicting effects, that is, the formation of silica without any nanostructure and an increasing degradation of the hexagonal lattice in the nanostructured parts (see Supporting Information, chapter 2), thus forbidding to draw a direct conclusion from them. The N_2_ physisorption isotherms and pore size distributions of **SBA‐15‐calc‐39MPa** and **SBA‐15‐as‐E‐p550‐39MPa** show only minor changes (Figure 4). The similarity is also reflected in the surface area and pore volume values listed in Table 1. The comparison between the unpressed (Figure 2) and the pressed samples (Figure 4) shows deviations within the range of error. This also applies to the comparison of **SBA‐15‐calc‐39MPa** and **SBA‐15‐as‐E‐p550‐39MPa**. Since the pore size distributions shown in Figure 4 as well as the SAXS curves in Figure 3 are very similar, we assume that the two template removal methods lead to the same mechanical stability of SBA‐15 against a pressure of 39 MPa.

**Figure 3:**
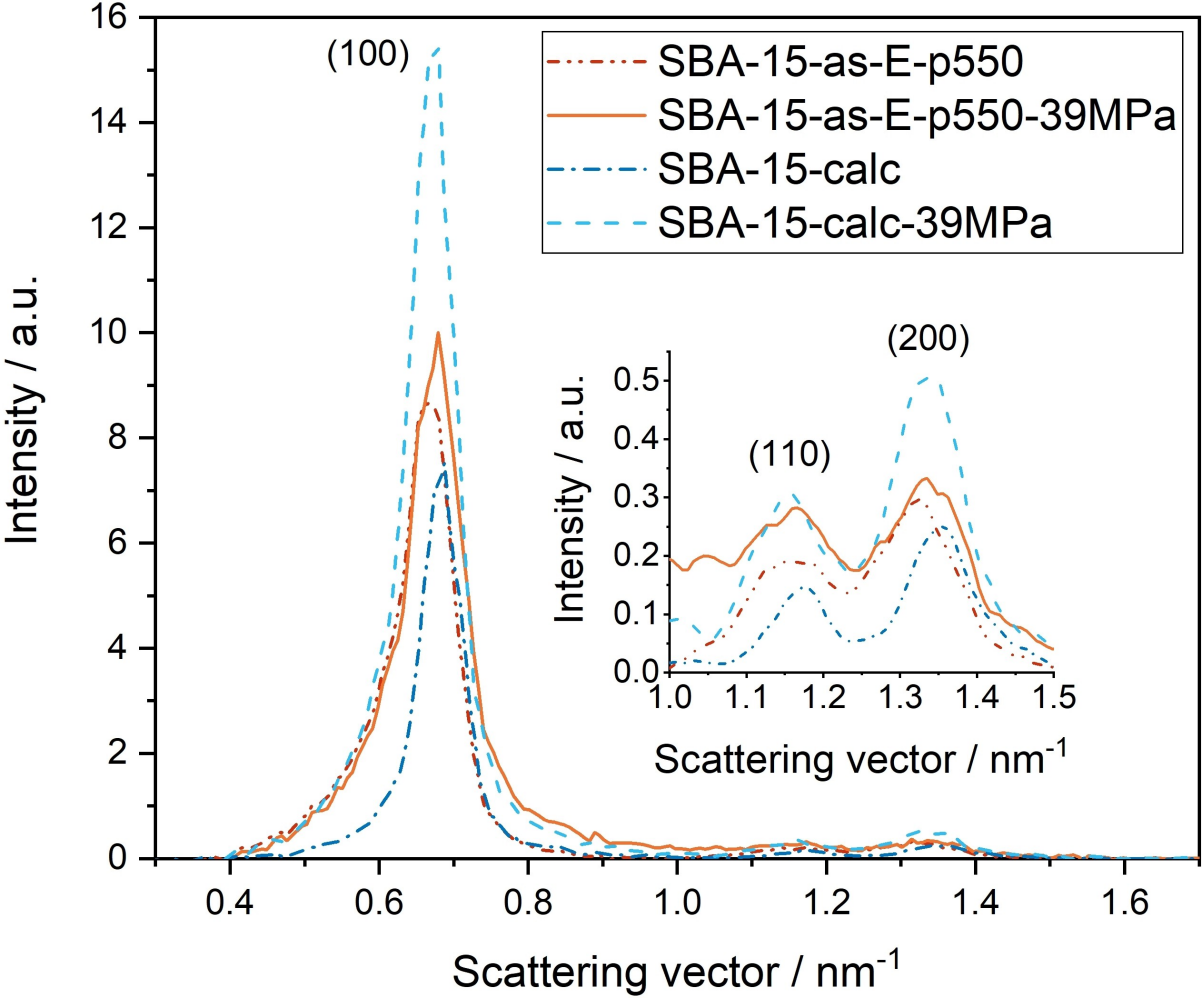
SAXS curves of **SBA‐15‐calc** and **SBA‐15‐as‐E‐p550** and their analogues pressed with 39 MPa for 10 min.

**Figure 4:**
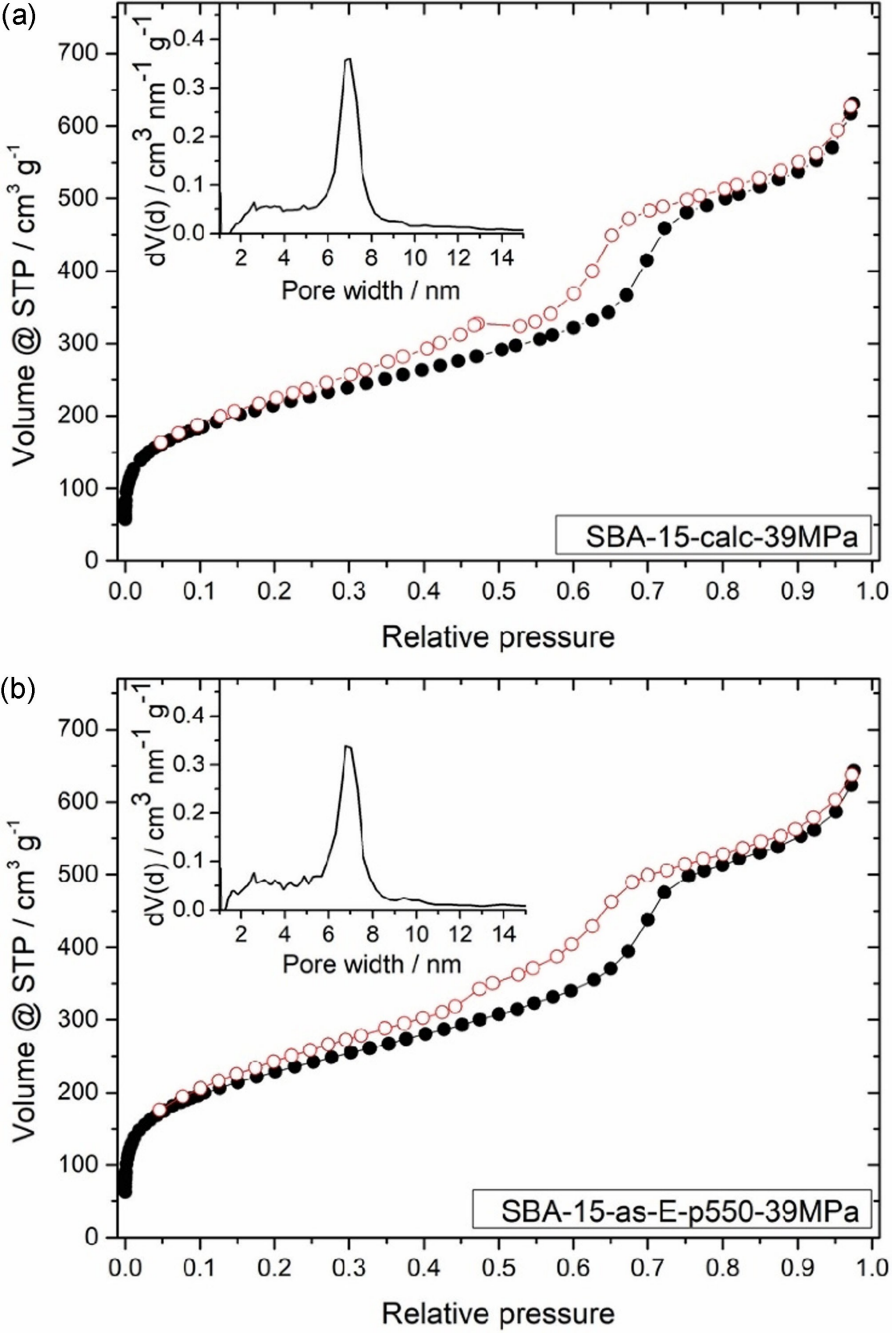
N_2_ adsorption (•) and desorption (○) isotherms as well as pore size distributions of (a) **SBA‐15‐calc‐39MPa** and (b) **SBA‐15‐as‐E‐p550‐39MPa**. The isotherms and the pore size distributions of the unpressed samples are depicted in Figure 2.

In conclusion, it is apparent that the specific template removal method influences the mechanical stability of SBA‐15. The comparison of the different methods shows that the thermal treatment of SBA‐15 has a positive influence on the mechanical stability of SBA‐15 against pressure. When the thermal treatment is performed in the presence of oxygen during the calcination, the mesoporous silica material is more stable against very high pressures, indicating that oxygen catalyzes the rearrangement of the silica network into a more stable conformation. However, if moderate pressures of up to 39 MPa are applied, SBA‐15, which was thermally treated in the presence of N_2_ at 550 °C, is just as stable as **SBA‐15‐calc**. Accordingly, solid catalysts from both routes of selective functionalization can be used for laboratory‐scale applications, with **SBA‐15‐as‐E‐p550** having the advantage of being 99 % template‐free and allowing for a more time‐ and material‐efficient selective functionalization.

## Conflict of interest

The authors declare no conflict of interest.

## Supporting information

As a service to our authors and readers, this journal provides supporting information supplied by the authors. Such materials are peer reviewed and may be re‐organized for online delivery, but are not copy‐edited or typeset. Technical support issues arising from supporting information (other than missing files) should be addressed to the authors.

Supporting Information
