# Supplementary material for: Influence of the Template Removal Method on the Mechanical Stability of SBA‐15
Source: ChemistryOpen. 2021 Nov 5;10(11):1123–8. doi: 10.1002/open.202100225 (PMC8569929; doi:10.1002/open.202100225)
Supplement: Supplementary file 1 — Supporting Information [file OPEN-10-1123-s001.pdf]

ChemistryOpen

Supporting Information

Influence of the Template Removal Method on the Mechanical Stability of SBA-15

Ann-Katrin Beurer, Johanna R. Bruckner,* and Yvonne Traa*

Table of Contents

1 Explanation of the Nomenclature 2
2 Mechanical Stability of SBA-15-calc 3
3 Experimental Section 7
4 Characterization Methods 9
5 SAXS Measurements 11
6 Influence of the Temperature during the Pretreatment in N_2 15
7 Literature 18

1 Explanation of the Nomenclature

For a better understanding of the present work, the nomenclature of the different samples is introduced in Table S1.

Table S1. Explanation of the sample nomenclature.

SBA-15-as	directly after the synthesis; called as-synthesized SBA-15
SBA-15-calc	pores were opened by calcination in air
SBA-15-calc-XMPA	pores were opened by calcination, and the sample was pressed with 8 to 156 MPa; abbreviated with “XMPa” depending on the pressure X
SBA-15-calc-re	pores were refilled with Pluronic® P-123; abbreviated with “re”
SBA-15-calc-re-E	pores were reopened with Soxhlet extraction with ethanol; abbreviated with an “E”
SBA-15-as-E	pores were opened with Soxhlet extraction with ethanol
SBA-15-as-E-p400	SBA-15-as-E pretreated in N_2 at 400°C; abbreviated with “p400”
SBA-15-as-E-p550	SBA-15-as-E pretreated in N_2 at 550°C; abbreviated with “p500”

2 Mechanical Stability of SBA-15-calc

To better understand if there is an influence of the template removal method on the mechanical stability of SBA-15 against pressure, the mechanical stability of SBA-15-calc was investigated first. Therefore, SBA-15-calc was pressed with pressures between 8 and 156 MPa. Afterwards, the different samples were investigated with SAXS and N_2 physisorption measurements.

The SAXS curves of the calcined and the pressed samples are pictured in Figure S1. All diffractograms shown have been normalized to the same intensity of the attenuated primary beam. Considering that the sample preparation and the measurement set-up were the same for every sample, this relative value depends on the X-ray absorption of the sample and the amount of scattered X-rays only.[1] The intensity of the attenuated beam *I* follows the equation

$$I = I_0 e^{-\mu x}, \qquad \text{(E1)}$$

with I_0 the intensity of the unattenuated primary beam, *x* the sample thickness and μ the linear attenuation coefficient. The latter relates to the total cross section σ_{tot} via

$$\sigma_{\text{tot}} = \mu / N_a, \qquad \text{(E2)}$$

with N_a the number of attenuating centers, i.e., atoms, per volume. This total cross section may be further divided into contributions from the photoelectric absorption (σ_{pe}), pair production (σ_{pp}), Compton (σ_{inel}) and Thomoson (σ_{el}) cross sections. Except for the Thomoson scattering, which arises due to the form or structure factor of the investigated sample, all of these contributions to the total cross section solely depend on the type and density of the atoms within the sample as well as the energy of the X-ray beam used. Taking into account that all samples consist of the same atoms, i.e., silicon and oxygen in a ratio of 1:2, differences in the scattering intensities after normalization to the attenuated primary beam may thus directly be attributed to a variation in the ratio of total silica to nanostructured silica.

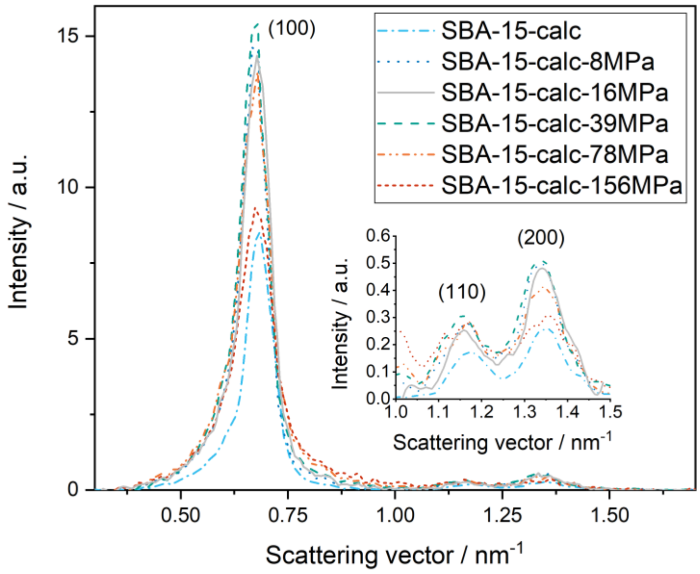

Figure S1. SAXS curves of SBA-15-calc and of SBA-15-calc-XMPa. The pressure was variated between 8 and 156 MPa.

As seen from Figure S1, the scattering intensity of SBA-15-calc-8MPa increases compared to the unpressed sample. This intensity increase indicates that the amount of unstructured silica is larger after applying pressure, which shows that a part of the SBA-15 is destroyed by this rather small pressure, already. At the same time, the full width at half maximum of the scattering peaks, which is plotted in Figure S2 for the example of the (100) reflection, stays constant, revealing that the properties of the nanostructured part of the sample stay unchanged. When increasing the pressure, the scattering intensities of the three characteristic peaks as well as their width stay almost constant up to a value of 39 MPa. When increasing the pressure to 78 MPa, the scattering intensity, especially of the (200) peak, starts to decrease, and the width of the peaks increases, a trend which continues for even higher pressures. This broadening of the scattering maxima was previously attributed to a reduction of the average diameter of the crystallites within the pressed sample.[2] However, if this was the case, the relative intensity of the individual peaks should not decrease,[3,4] but rather increase as the amount of unstructured silica rises further. Thus, it seems more likely to us that the observed broadening is due to an increasing disorder of the second kind, which means that the positions of the hexagonally arranged pores become more and more “blurred”.[5] Such blurring may arise, if parts of the pores collapse, leading to plugged or partially filled pores. Chytil et al.[6] came to the same conclusion in their work.

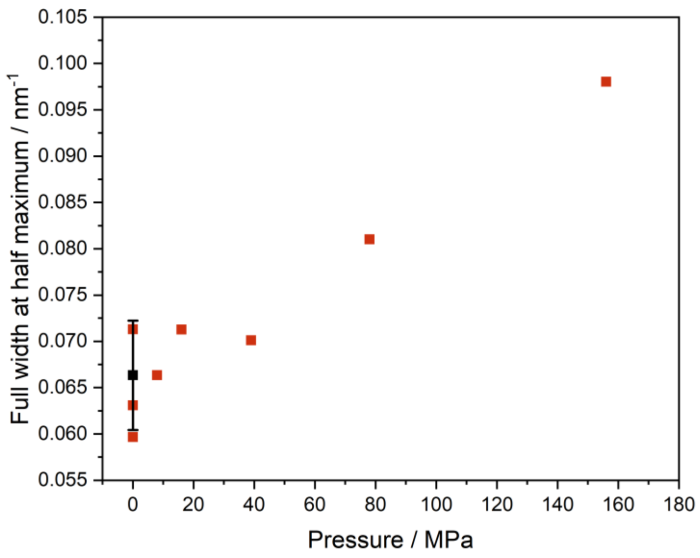

Figure S2. Full width at half-maximum *w* of the (100) peak from the SAXS curves of calcined SBA-15 plotted against the applied pressure. The black data point at 0 MPa depicts the average over four individual measurements, indicating the accuracy of the method.

In addition to the SAXS measurements, N_2 physisorption measurements were performed. The resulting isotherms are shown in Figure S3(a). Unpressed SBA-15-calc shows the typical type IV isotherm.[7] With increasing pressure, the type of the isotherms changes, and the hysteresis gets smaller. This is an indication for a loss of mesoporosity within SBA-15-calc.[8] The results evaluated from the N_2 physisorption measurements are listed in Table S2. The decrease in surface area and pore volume, as described in the literature and expected from the change in isotherms of the N_2 physisorption measurements, are not so pronounced. The comparison of the pore size distribution of the different samples shows a change with increasing pressure (Figure S3(b)). It can be seen that the amount of pores with the main pore diameter decreases with increasing pressure.[6,7] This leads to the conclusion that SBA-15-calc is mechanically stable against pressures up to 39 MPa. At higher pressures, there is an increasing loss of the hexagonal structure of SBA-15.

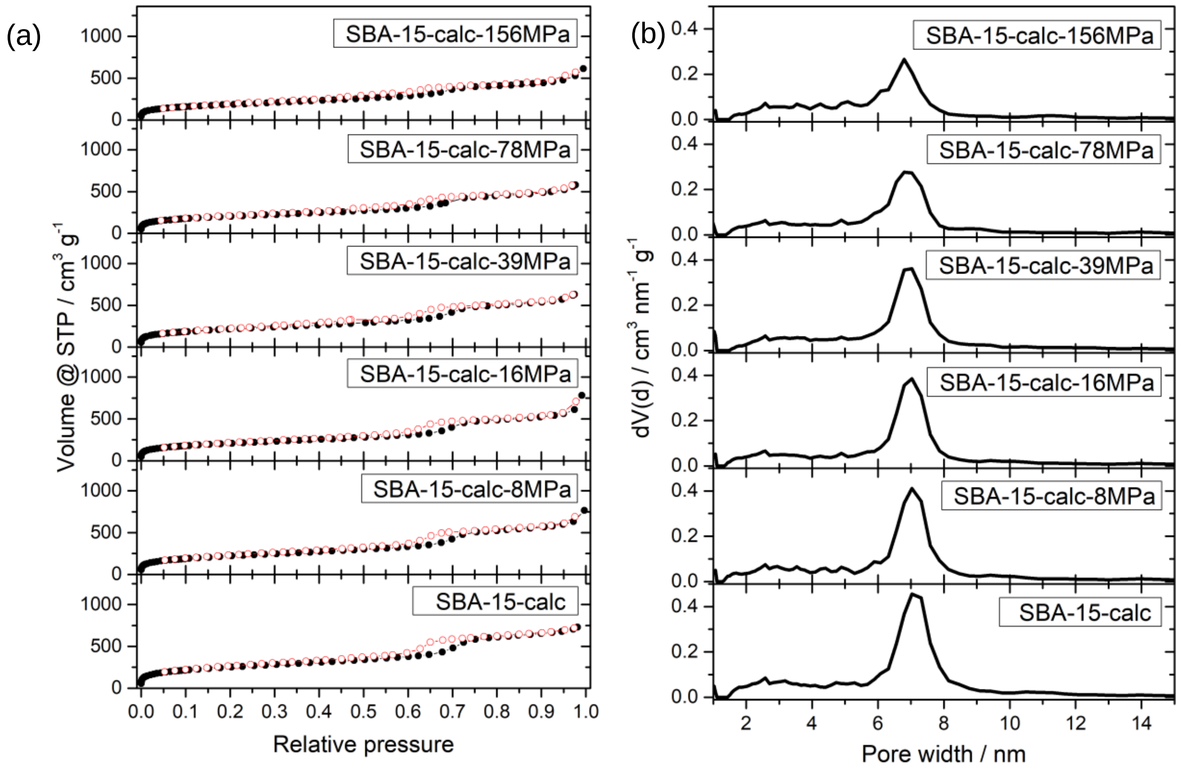

Figure S3. (a) Adsorption (●) and desorption (○) isotherms and (b) pore size distributions of SBA-15-calc and of SBA-15-calc-XMPa. The pressure was variated between 8 and 156 MPa.

Table S2. The total surface determined by the BET method (S_{BET}) and micropore surface (S_{micro}) as well as the total pore volume (V_{tot}) and the micropore volume (V_{micro}) of SBA-15-calc and of SBA-15-calc-XMPa samples are listed. The pressure was variated between 8 and 156 MPa. Furthermore, the pore diameters determined by the DFT method ($d_{pore,DFT}$) are given.

Sample name	S_{BET} / m^2 g^{-1}	S_{micro} / m^2 g^{-1}	V_{tot} / cm^3 g^{-1}	V_{micro} / cm^3 g^{-1}	$d_{pore,DFT}$ / nm
SBA-15-calc	897	222	1.128	0.095	7.0
SBA-15-calc-8MPa	800	195	1.186	0.076	7.0
SBA-15-calc-16MPa	750	208	1.209	0.085	7.1
SBA-15-calc-39MPa	878	258	1.101	0.106	6.8
SBA-15-calc-78MPa	723	208	0.897	0.089	6.8
SBA-15-calc-156MPa	670	125	0.948	0.049	6.8

3 Experimental Section

Synthesis of SBA-15

For the synthesis of SBA-15, 16 g of triblock copolymer Pluronic® P-123 (average molar mass ~ 5800 g mol^{-1}, Sigma Aldrich) was dissolved in a mixture of 520 ml demineralized water and 80 ml 37 wt% hydrochloric acid at room temperature at a stirring speed of 100 rpm overnight in a 1 l teflon-coated autoclave (Berghof Products + Instruments GmbH). The solution was heated to 45°C before adding 37 ml tetraethyl orthosilicate (TEOS, 98 %, reagent grade, Sigma Aldrich). The mixture was stirred for 7.5 h at 45°C with a stirring speed of 150 rpm. A hydrothermal treatment under static conditions at 80°C followed. Afterwards, the SBA-15-as was separated under vacuum, washed with demineralized water and dried in an oven at 80°C. The SBA-15-as of one batch was used as starting material for all experiments in this work.

Removal of Pluronic® P-123 – Calcination

To open the pores, SBA-15-as was calcined for 6 h at 550°C in an air flow of 150 l h^{-1}. The heating rate was 1 K min^{-1}.

Refilling of the Pores

To refill the pores with template, Pluronic® P-123 was dissolved in ethanol overnight. SBA-15-calc was added to the solution, and the suspension was stirred for 42 h at room temperature. The solution used to refill the pores was 4 g Pluronic® P-123 in 60 ml ethanol per gram calcined SBA-15. Subsequently, the SBA-15 with refilled pores (SBA-15-calc-re) was separated under vacuum, washed with 500 ml of demineralized water and dried in an oven at 80°C.

Removal of Pluronic® P-123 – Soxhlet Extraction

The triblock copolymer Pluronic® P-123 was removed from the pores of SBA-15-as or SBA-15-calc-re by Soxhlet extraction for 112 h using ethanol as extracting agent. The extracted materials (SBA-15-as-E and SBA-15-calc-re-E) were dried in an oven at 80°C.

Pretreatment in N_2

SBA-15-as-E was treated in an oven at 400°C or 550°C for 6 h in N_2. The treatment was performed with a heating rate of 2°C min^{-1} and a N_2 flow of 58 l h^{-1}. The product obtained was named SBA-15-as-E-p400 or SBA-15-as-E-p550 depending on the temperature during the pretreatment.

Investigation of the Mechanical Stability

For the investigation of the mechanical stability, the press FluXana (Vaneox® Pressing Technology) was used. For each experiment, 150 mg of the respective sample was pressed with different pressures. The pressed tablets were then carefully crushed again in a mortar in order to be able to characterize the samples.

4 Characterization Methods

Small angle X-ray scattering (SAXS)

The powdery samples were filled into mark capillaries with a diameter of 1 mm (Hilgenberg, glass no. 14) and flame-sealed. For measurements, a SAXSess mc^2 diffractometer (Anton Paar) in the line collimation geometry was used for which the sample to detector distance was calibrated with cholesteryl palmitate. X-ray radiation with a wavelength of $\lambda(\text{Cu-K}\alpha) = 0.1542$ nm was generated by an ID 3003 X-ray generator (Seifert) operated at 40 kV and 40 mA. The sample housing was evacuated prior to measurements, which were carried out at 25°C with the help of a TCS 120 hot stage (Anton Paar) and averaged over 60 individual measurements. The scattered X-ray intensity was detected with a one-dimensional CMOS Mythen 2K detector (Dectris). The semi-transparent beam-stop allowed for an additional measurement of the transmitted primary X-ray beam. Using the software SAXSquant™, the measured scattering profiles were normalized to the same intensity of the attenuated primary beam, background-corrected and deconvoluted. Subsequently, the scattering curves were Lorentz- and polarization-corrected. The scattering associated to the form factor of the mesopores as well as further incoherent scattering was removed by fitting and subtracting a double exponential decay, leaving only the part of the scattering, which originates from the hexagonal structure. The obtained Bragg-like diffraction maxima were fitted with Lorentzian functions to extract the exact peak positions and full widths at half maximum.

N_2 physisorption

The surface area as well as the pore size of the SBA-15 samples were analyzed by N_2 physisorption measurements. The adsorption and desorption isotherms were recorded using Autosorb 3B from Quantachrome Instruments. Before the measurements, the samples were outgassed under vacuum at 200°C for 16 h. After the pretreatment, the N_2 physisorption measurements were performed in a liquid N_2 bath at −196°C. From the adsorption isotherms, the surfaces were calculated using the BET method, whereas the pore sizes and pore size distributions were determined with the DFT method, taking into account the hexagonal structure.

Elemental analysis

The amount of carbon and hydrogen was measured with an Elemental Analizer 1106 from the company Carlo Erba Strumentazione.

The results of the elemental analysis (Table S3) show how much of the structure-directing template was removed from the pores during the pore opening. With these results, it is possible

to calculate the percentage of removed Pluronic® P-123 *(Δ)* for the different samples. The calculation for SBA-15-calc follows the equation

$$\Delta = 100 \cdot \left(1 - \frac{w(\mathrm{C\ in\ SBA{-}15{-}calc})}{w(\mathrm{C\ in\ SBA{-}15{-}as})}\right) = 100 \cdot \left(1 - \frac{0.26\ wt.\%}{8.90\ wt.\%}\right) = 97\ \% \qquad \text{(E3)}$$

Depending on the template removal method of the structure-directing template Pluronic® P-123, the corresponding starting material is used for the calculation. For the calculation of the removed amount of template of SBA-15-as-E, SBA-15-as-E-p400 and SBA-15-as-E-p550, the carbon amount of SBA-15-as is used as starting material. The amount of removed Pluronic® P-123 for SBA-15-calc-re-E was calculated from the carbon amount of SBA-15-calc-re. The calculated results are listed in Table S3.

Table S3. Results of the elemental analysis and the calculated percentage of the removed structure-directing template (Δ). The percentage of the removed template was calculated from the value of the starting material of the respective method for removing the template.

Sample name	*w*(C) / wt.%	*w*(H) / wt.%	*Δ* / %
SBA-15-as	8.90	2.50	-
SBA-15-calc	0.26	0.89	97
SBA-15-calc-re	21.37	3.52	-
SBA-15-calc-re-E	8.03	1.86	62
SBA-15-as-E	7.60	2.80	15
SBA-15-as-E-p400	0.10	1.00	99
SBA-15-as-E-p550	0.09	1.02	99

5 SAXS Measurements

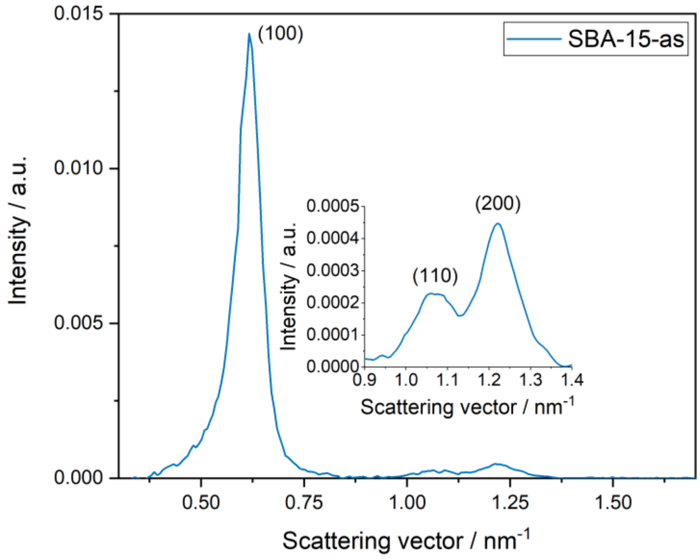

Figure S4. SAXS curve of SBA-15-as.

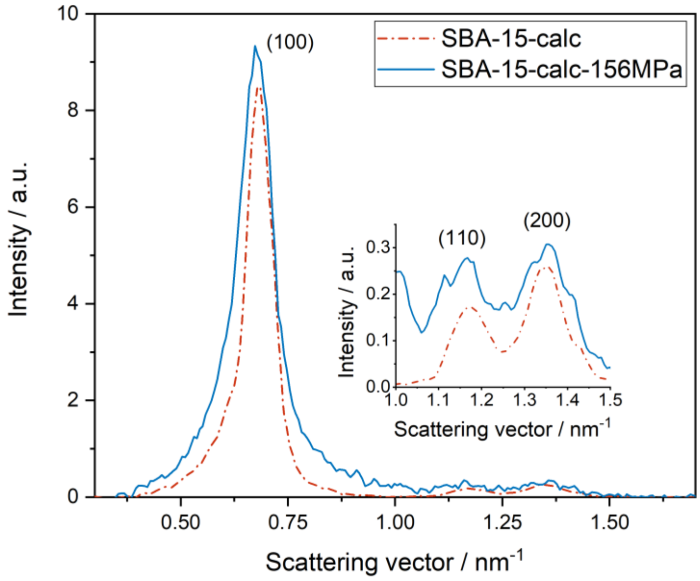

Figure S5. SAXS curves of SBA-15-calc prior and after applying a pressure of 156 MPa for 10 min. The increase of the full width at half maximum is 48 %.

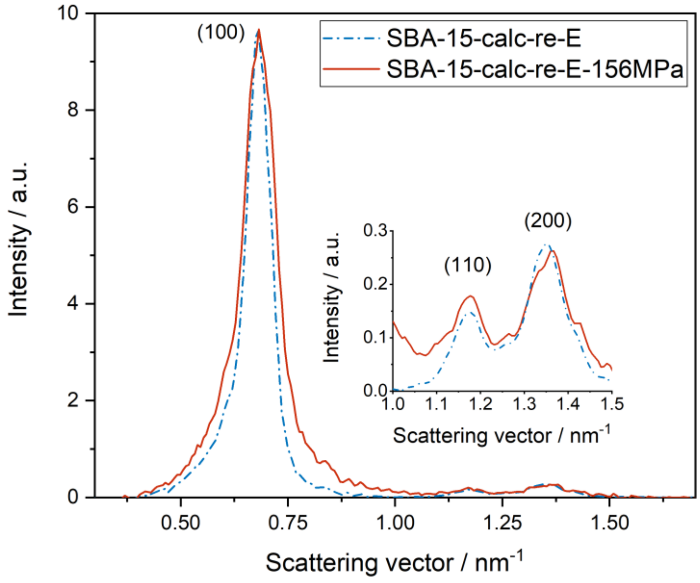

Figure S6. SAXS curves of SBA-15-calc-re-E prior and after applying a pressure of 156 MPa for 10 min. The increase of the full width at half maximum is 38 %.

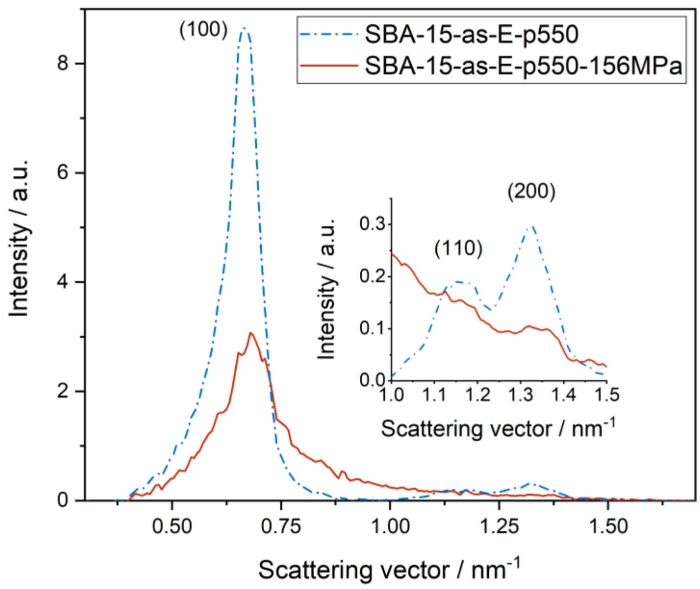

Figure S7. SAXS curves of SBA-15-as-E-p550 prior and after applying a pressure of 156 MPa for 10 min. The increase of the full width at half maximum is 72 %.

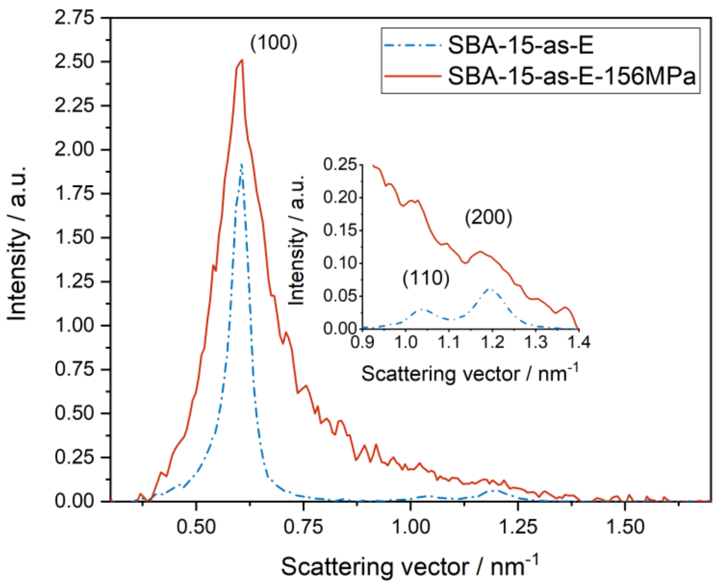

Figure S8. SAXS curves of SBA-15-as-E prior and after applying a pressure of 156 MPa for 10 min. The increase of the full width at half maximum is 190 %.

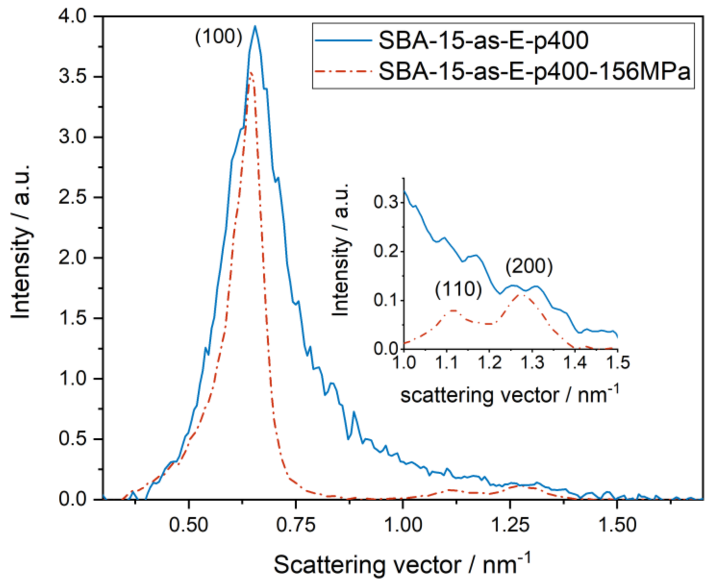

Figure S9. SAXS curves of SBA-15-as-E-p400 prior and after applying a pressure of 156 MPa for 10 min. The increase of the full width at half maximum is 94 %.

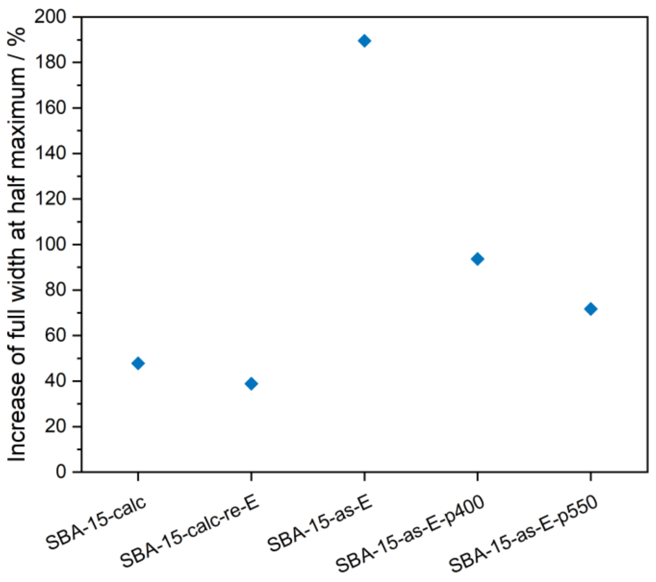

Figure S10. Percentage increase of the full width at half maximum (FWHM) of Lorentzian function fitted to the (100) peak upon pressing.

6 Influence of the Temperature during the Pretreatment in N_2

As described in the main article, SBA-15-as-E was pretreated at 400°C and 550°C in N_2 and subsequently analyzed by elemental analysis, SAXS measurements, and N_2 physisorption measurements.

Observation of the calculated fractions of removed Pluronic® P-123 (Table S3) shows that after thermal treatment at 400°C and 550°C, the remaining template was removed from the pores and all pores were accessible.

The SAXS curves of SBA-15-as-E-p400 in Figure S9 and of SBA-15-as-E-p550 in Figure S7 are almost identical and show the characteristic reflections (100), (110) and (200). However, the comparison to the SAXS curves of the pressed samples SBA-15-as-E-p400-156MPa (Figure S9) and SBA-15-as-E-p550-156MPa (Figure S7) show some differences. In particular, while reflections (100) and (200) are not visible in the SAXS curve of SBA-15-as-E-p400-156MPa, the reflections in the SAXS curve of SBA-15-as-E-p550-156MPa can be adumbrated. Therefore, it can be assumed that SBA-15-as-E-p550 was not damaged to the same extent as SBA-15-as-E-p400 and, thus, higher temperatures during pretreatment in N_2 have a positive effect on the mechanical stability of SBA-15 against pressure. Furthermore, a decrease of the lattice parameter with increasing temperature can be observed compared to SBA-15-as, due to the pretreatment in N_2 (Table S4). This shrinkage is accompanied by a shrinkage of the pores (Table S4).

These results are confirmed by the N_2 physisorption measurements. While the shapes of the N_2 physisorption isotherms and their relatively narrow pore size distribution of the two unpressed samples are very similar, the samples pressed at 156 MPa differ significantly from the unpressed materials (Figure S11). Looking at the different surface areas, pore volumes and pore sizes determined from the N_2 physisorption measurements for SBA-15-as-E-p400 and SBA-15-as-E-p550 in Table S4, smaller differences are seen. This can be explained by the stronger shrinkage of the pores at higher temperatures. It is known from the literature that the unit cell shrinks at higher temperatures in air due to further condensation of the silica framework.[9] It seems that the oxygen in the air as well as from desorbed water molecules acts as catalyst for reordering the silica bonds in the lattice.[10] However, it is also known, that the temperature range in which the strongest shrinkage is observable is between 300°C and 500°C.[9] Accordingly, it can be assumed that a similar effect is also obtained when heating in N_2, so that further condensation reactions make the pore walls more ordered and thus more stable to pressure. In this work, the pretreatment is done in N_2 at 400°C and at 550°C, and the shrinkage is more pronounced for SBA-15-as-E-p550. This leads to the assumption that the higher temperature is necessary to overcome the binding energies to reorder the silica lattice.

This leads to a more ordered silica lattice and results in more stable pore walls for SBA-15-as-E-p550 compared to SBA-15-as-E-p400.

A comparison of the unpressed materials with those pressed at 156 MPa shows large differences between the N_2 physisorption isotherms and pore size distributions. The analysis shows the expected decrease in surface areas and pore volumes due to pressing at 156 MPa. In addition, the comparison of SBA-15-as-E-p400-156MPa and SBA-15-as-E-p550-156MPa shows further differences. Although the two N_2 physisorption isotherms look similar, large differences between the pore size distributions are evident. While the amount of the original main pores has almost completely disappeared for SBA-15-as-E-p400-156MPa, it has also decreased for SBA-15-as-E-p550-156MPa, but is still perceived as the main fraction. Accordingly, the pore structure of SBA-15-as-E-p550-156MPa was less damaged. This confirms the assumptions of the SAXS studies and shows that a higher temperature during the pretreatment has a positive effect on the mechanical stability of SBA-15 against pressure.

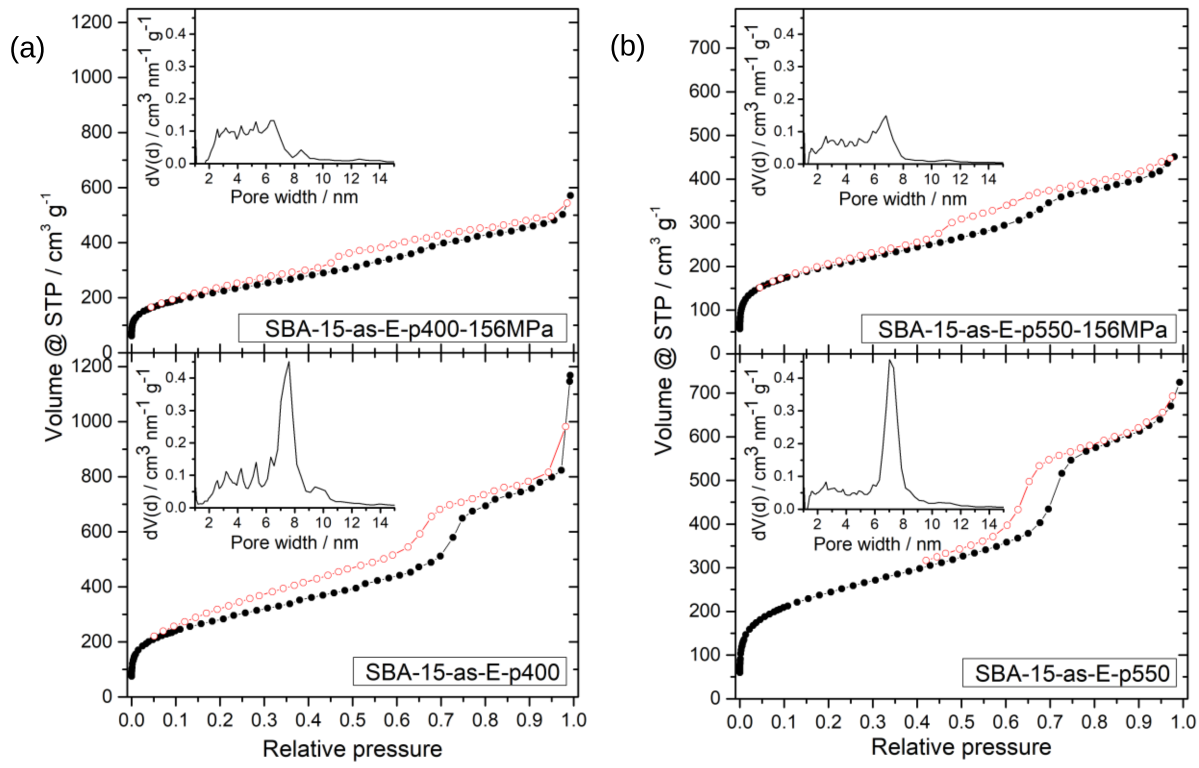

Figure S11. Adsorption (●) and desorption (○) isotherms and pore size distributions of (a) SBA-15-as-E-p400 and (b) SBA-15-as-E-p550 and their analogues pressed with 156 MPa for 10 min.

Table S4. Total surface determined by the BET method (S_{BET}), micropore surface (S_{micro}) as well as the total (V_{tot}), the mesopore (V_{meso}) and the micropore volume (V_{micro}) of SBA-15-as-E-p400, SBA-15-as-E-p550 and their analogues pressed with 39 or 156 MPa. Furthermore, the pore diameters determined by the DFT method ($d_{pore,DFT}$) and the lattice parameter (a) determined from SAXS are listed.

SBA-15-	S_{BET} / m^2 g^{-1}	S_{micro} / m^2 g^{-1}	V_{tot} / cm^3 g^{-1}	V_{meso} / cm^3 g^{-1}	V_{micro} / cm^3 g^{-1}	$d_{pore,DFT}$ / nm	a / nm
as	-	-	-	-	-	-	11.9
as-E-p400	1016	140	1.807	1.751	0.056	7.6	11.4
as-E-p400-156MPa	798	132	0.884	0.830	0.054	6.6	11.3
as-E-p550	869	236	1.148	1.053	0.095	7.0	11.1
as-E-p550-39MPa	819	222	0.995	0.902	0.093	6.8	11.0
as-E-p550-156MPa	720	209	0.697	0.610	0.087	6.8	10.8

7 Literature

[1] J. H. Hubbell, S. M. Seltzer, "Tables of X-Ray Mass Attenuation Coefficients and Mass Energy-Absorption Coefficients". National Institute of Standards and Technology (NIST). Retrieved 14 July 2021.

[2] M. Hartmann, A. Vinu, *Langmuir* **2002**, *18*, 8010-8016.

[3] P. Scherrer, *Nachrichten von der Gesellschaft der Wissenschaften zu Göttingen, Mathematisch-Physikalische Klasse* **1918**, 98-100.

[4] C. F. Holder, R. E. Schaak, *ACS nano* **2019**, *13(7)*, 7359-7365.

[5] R. Hosemann, *Z. Physik* **1950**, *128(4)*, 465-492; R. Hosemann, S. N. Bacchi, *Phys. Rev.* **1954**, *94(1)*, 71-74; R. Hosemann, P. H. Lindenmeyer, *J. Appl. Phys.* **1963**, *34(1)*, 42-45.

[6] S. Chytil, L. Haugland, E. A. Blekkan, *Microporous Mesoporous Mater.* **2008**, *111*, 134-142.

[7] V. Meynen, P. Cool, E. F. Vansant, *Microporous Mesoporous Mater.* **2009**, *125*, 170-223.

[8] M. Thommes, K. Kaneko, A. V. Neimark, J. P. Olivier, F. Rodriguez-Reinoso, J. Rouquerol, K. S. W. Sing, *Pure Appl. Chem.* **2015**, *87(9-10)*, 1051-1069.

[9] F. Kleitz, W. Schmidt, F. Schüth, *Microporous Mesoporous Mater.* **2003**, *65*, 1–29.

[10] T. Tatsumi, K. A. Koyani, Y. Tanaka, S. Nakata, *J. Porous Mater.* **1999**, *6*, 13-17.
